# Outcomes of pregnancy-related acute kidney injury: A retrospective study in the obstetric critical care unit at Kenyatta National Hospital 2020 to 2023

**DOI:** 10.1371/journal.pgph.0004396

**Published:** 2025-04-08

**Authors:** Wanjiku Ng’ethe, Anne Pulei, Diana Ondieki, James Amenge, Rose Kosgei, Joshua Kayima, Alfred Osoti

**Affiliations:** 1 Department of Obstetrics and Gynaecology, University of Nairobi, Nairobi, Kenya; 2 Department of Obstetrics and Gynaecology, Kenyatta National Hospital, Nairobi, Kenya; 3 Department of Internal Medicine, University of Nairobi, Nairobi, Kenya; Centre of Biomedical Ethics and Culture, PAKISTAN

## Abstract

Pregnancy-related acute kidney injury (PrAKI) is defined as a rapid decline in kidney function in the pregnancy or puerperal period that can result in life-threatening organ dysfunction. This study aimed to investigate socio-demographic features of critically ill women with pregnancy-related acute kidney injury as well as their maternal and foetal outcomes. Retrospective analysis of data in patients with pregnancy-related kidney injury in the obstetric critical care unit at a public tertiary referral centre in Kenya between February 2020–2023. Of the 266 patient files reviewed, pregnancy-related acute kidney was found in 203 patients. The main predisposing factors for acute kidney injury were hypertensive disorders in pregnancy (64.1%), obstetric haemorrhage (38.4%) and sepsis (36.5%). According to KDIGO (Kidney Disease Improving Global Outcomes) criteria, 44 patients presented in stage 1 (21.7%), 58 in stage 2 and 101 in stage 3 (49.8%). Patients with KDIGO stage 3 had a higher risk of high SOFA (Sequential Organ Failure Assessment) score (*p* =< 0.001), longer ICU stay (*p* = 0.008) and longer duration on ventilation (*p* = 0.010). Seventy-six patients underwent dialysis (37.4%). Recovery of renal function was complete in 91 patients (44.8%), partial in 41 (20.2%) with dependence on dialysis seen in 23 (23.6%). Forty-eight patients died (23.6%). Risks associated with mortality were mechanical ventilation (*p =* 0.001) and inotropic support (*p =*< 0.001) with statistically significant higher mean SOFA scores in those who died versus those who survived (12.6 ± 3.8 *p =<* 0.001 vs 8.6 ± 3.2). The incidence of PrAKI is still underestimated in the Kenyan setting with the majority of the patients presenting with advanced renal injury. These patients are at higher risk of adverse maternal morbidity and mortality in the critical care setting.

## Introduction

Pregnancy-related acute kidney injury (PrAKI) is defined as a rapid decline in kidney function in the pregnancy or puerperal period that can result in life-threatening organ dysfunction [1–4]. Acute kidney injury is increased 0.5 fold in pregnancy due to maternal predisposing factors; hypertensive disorders of pregnancy, sepsis and obstetric haemorrhage [5,6]. Once considered a rare sequela of pregnancy in developing countries, there has been a rise in PrAKI due to a shift in the epidemiological landscape of pregnant women attributed to older age, obesity, pre-existing conditions and the advent of assisted reproductive technology [7,8]. Research on PRAKI in developing countries has been minimal as noted in a systematic review of the burden of disease in Africa [9]. The earliest study in South Africa in 1995 showed a significant change in the aetiology with a decline in gynaecological and a significant increase in obstetric-related causes (p = 0.0003) [10]. Currently, it is estimated that PrAKI affects ~1 in 1000 deliveries in Africa with pregnancy representing 16% of all causes largely due to late detection, late referral and lack of infrastructure in nephrology [9–11]. It represents a major public health concern with an increased risk of adverse maternal and foetal outcomes including the need for ICU admission, dependence on dialysis, and maternal and perinatal mortality [12–14].

Despite the increased burden that PrAKI poses on the health systems of developing nations, there is limited data from Africa. The systematic review of PrAKI in Africa showed only 14 studies conducted between 1995–2021 correlated PrAKI to maternal outcomes with the main predisposing factors of preeclampsia, haemorrhage and sepsis showing not much change in the 26 years of the review. Only 8 included foetal outcomes, while only 2 investigated critically ill obstetric patients [9]. Locally, the prevalence rate of PrAKI in the antenatal setting is estimated at 3.2%(n = 66 N = 2068) with 19 of those dialyzed and no documented mortality [15]. Preeclampsia and HELLP syndrome (Haemolysis Elevated Liver Enzymes Low Platelets) were found to be a major cause of AKI, however, neither study was conducted in the ICU setting [15–17].

This study aimed to investigate socio-demographic features of pregnancy-related acute kidney injury as well as their maternal and foetal outcomes within the critical care setting.

## Methods

This retrospective descriptive study was conducted between February 2020–2023 on patients with pregnancy-related acute kidney injury at the 5-bed obstetric critical care unit in Kenyatta National Hospital (KNH), the largest tertiary referral hospital in Kenya. All files of pregnant women admitted to the maternity ICU with the International Classification of Diseases (ICD 10) diagnosis of Pregnancy-related Acute Kidney Injury (266 patients) were recruited into the study. Acute kidney injury was defined by Kidney Disease Improving Global Outcomes (KDIGO) criteria. Patients found to have PRAKI (203 patients) were included in the study and classified into stages I, II and III on admission to ICU. Files of patients with a history of renal transplant as well as those with missing and incomplete data were excluded.

A study done in a 10-bed maternity ICU in Brazil showed a prevalence of PRAKI of 27.8% with a precision of 0.05 [12]. The sample size calculation of 308 was adjusted for a finite population and a difference in bed capacity, resulting in a minimum sample size of 203. Consecutive sampling technique was used for the retrieval of the files.

Data recorded were: demographic data, obstetric history, clinical characteristics, comorbidities, KDIGO stage, cause of AKI and vital signs, Sequential Organ Failure Assessment (SOFA) score which was assessed within 24 hours of admission, number of patients who underwent dialysis and their indications, critical care therapeutic management (transfusion of blood and blood products, use of mechanical ventilation and inotropic drugs), maternal and foetal outcomes, length of ICU stay and maternal mortality. Hypertensive disorders of pregnancy were defined by the American College of Obstetricians and Gynaecologists [18]. Organ dysfunction was calculated using the SOFA scoring system using the patient’s FiO2 requirement, inotropic dosage, serum creatinine, platelet count and Glasgow Coma Scale.

Haemodialysis initiation was after Nephrology review of urine output, kidney function tests and triple serology (HIV, Hepatitis B and C) results. Recovery from acute kidney injury was divided into partial and complete; 14 days from the time of diagnosis. Complete recovery was defined as a return to baseline creatinine while partial recovery was defined as deranged kidney function no longer requiring RRT.

Data collection began on 16th March 2023 and was completed on 30th March 2023 and was done within the records department of KNH after approval from the Head of Department, Obstetrics and Gynaecology. Files drawn were for patients admitted from 1st February 2020–1st February 2023. The variables were then inputted into the REDCAP software for analysis by the statistician using SPSS 26. Descriptive statistics such as frequencies, means and standard deviations were used for continuous variables while for categorical variables frequencies with their percentages were tabulated. Mortality was calculated as a proportion and presented as a percentage. For all statistical tests, a p-value of <0.05 was considered statistically significant.

### Ethical statement

Ethical approval was sought from KNH-UON ethics and Research Committee requirements (Protocol 813), study number P775/10/2022. Approvals to collect data were sought from the KNH Department of Research and Department of Obstetrics and Gynaecology, University of Nairobi. Patient confidentiality was assured by using study numbers and uploaded to a secure encrypted server. The primary author (myself) had access to the patient’s hospital numbers. On RedCap Software the identifiers were study numbers as seen in S1 Data which were then handed to the data analyst. Consent was not sought from the patients for anonymity.

## Results

**Fig 1:**
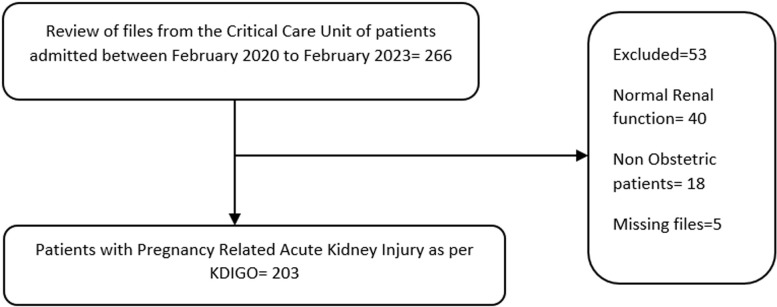
Obstetric patients diagnosed with pregnancy-related acute kidney injury in the obstetric ICU.

Of the 266 patient files reviewed from the maternity Critical Care Unit, 203 patients were found to have the diagnosis of pregnancy-related acute kidney injury as per KDIGO guidelines between February 2020–2023 as shown in Fig 1: Obstetric patients diagnosed with pregnancy-related acute kidney injury in the obstetric ICU. 40 were found to have normal renal function tests, 18 were non-obstetric and 5 files had incomplete data as per the study flow chart.

As shown in Table 1, the median age was 28.0 (IQR 22.0 – 33.0) years. Majority of the participants were aged between 26.0 to 35.0 years (47.3%), were referrals (95.1%), resided in urban areas (64.5%), had a secondary level of education (58.1%), were multi-para (61.1%), were in gestation of 33–37 weeks at the time of delivery (33.0%), and had attended less than 4 ANC visits (79.3%). Majority of the deliveries were by Caesarean section (n = 120,59.1%).

**Table 1:** Characteristics of patients with PrAKI patients.

	Frequency *(n = 203)*	Percent
**Age in years**		
<18	8	3.9
18 – 25	68	33.5
26 – 35	96	47.3
>35	31	15.3
**Referral**		
Yes	193	95.1
**Residence**		
Urban	131	64.5
**Level of education**		
Informal	8	3.9
Primary	54	26.6
Secondary	118	58.1
Tertiary	23	11.3
**Gravidity**		
1	52	25.6
2 – 4	124	61.1
≥5	27	13.3
**Gestation in weeks**		
<28	36	17.7
28 – 32	53	26.1
33 – 37	67	33.0
>37	47	23.2
**ANC visits**		
<4	161	79.3
≥4	42	20.7
**Comorbidities**		
Chronic HTN	14	6.9
Anaemia	153	75.4
Pre-existing kidney disease	13	6.4
**Maternal predisposing factors**		
Multiple gestation	13	6.4
Preeclampsia	45	22.2
Bladder injury	5	2.5
APH	26	12.8
Eclampsia	85	41.9
Diabetic ketoacidosis	10	4.9
PPH	52	25.6
H. Gravidarum	2	1.0
Sepsis	74	36.5
DIC	20	9.9
HELLP	66	32.5

*ANC* Antenatal clinic *HTN* Hypertension *APH* Antepartum Haemorrhage *PPH* Postpartum Haemorrhage.

*H. Gravidarum* Hyperemesis gravidarum *DIC* Disseminated Intravascular Coagulopathy *HELLP* Haemolysis.

Elevated Liver Enzymes Low Platelets.

¶ Patients referred from other medical facilities for specialized care.

† Gestation at the time of delivery

The major comorbidity was anaemia n = 153 (75.4%) with equal numbers of moderate and severe anaemia (n = 64 respectively) while chronic hypertension and pre-existing kidney disease showed a very small percentage. The main causes of AKI were hypertensive disorders of pregnancy n = 130 (preeclampsia n = 45, 22.2% and eclampsia n = 85,41.9%), Obstetric Haemorrhage n = 78 (Antepartum Haemorrhage n = 26, 12.8% and Postpartum Haemorrhage n = 52, 25.6%) and Sepsis n = 74,36.5%. Majority of the deliveries were via Caesarean section (n = 120, 59.1%).

**Fig 2:**
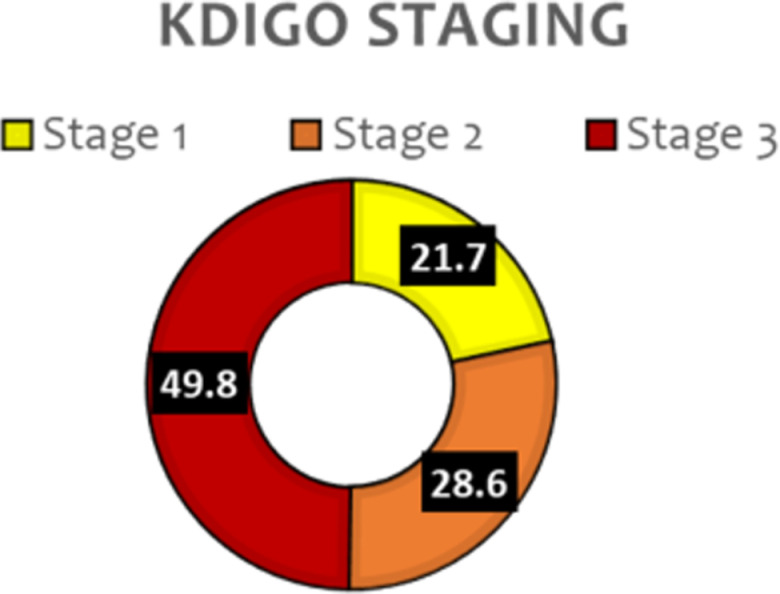
Severity of AKI as per KDIGO staging.

The severity of AKI staging as per KDIGO is shown in Fig 2: Severity of AKI as per KDIGO staging. Stage 1 (n = 44, 21.7%), Stage 2 (n = 58,28.6%) with majority in Stage 3 (n = 101, 49.8%). The means for the SOFA scores, days spent in ICU, and number of days on ventilation for the 3 stages were statistically different, however, stage 3 had a higher risk of high SOFA score (11.2 ± 3.6 *p* =< 0.001), longer ICU stay (7.5 ± 7.4 *p* = 0.008) and longer duration on ventilation (5.5 ± 7.7 *p* = 0.010) as shown in Table 2 below. There was no statistical difference with the foetal outcomes of the 3 KDIGO stages.

**Table 2:** KDIGO severity with selected patient characteristics.

	KDIGO severity			p-value
	1 *(n = 44)*	2 *(n = 58)*	3 *(n = 101)*	
**SOFA score,** *Mean ± SD*	7.6 ± 2.7	8.1 ± 3.6	11.2 ± 3.6	**<0.001**
**ICU days,** *Mean ± SD*	4.9 ± 4.0	4.8 ± 4.0	7.5 ± 7.4	**0.008**
**Ventilation days,** *Mean ± SD*	2.7 ± 3.9	2.3 ± 2.2	5.5 ± 7.7	**0.010**
**Maternal outcome,** *n (%)*				
Complete recovery of renal function	39 (88.6)	37 (63.8)	15 (14.9)	**<0.001**
Partial recovery of renal function	3 (6.8)	10 (17.2)	28 (27.7)	
Dependence on dialysis	0 (0.0)	0 (0.0)	23 (22.8)	
Death/Maternal mortality	2 (4.5)	11 (19.0)	35 (34.7)	
Foetal outcome*, *n (%)*				
Miscarriage	3 (6.8)	9 (15.5)	16 (15.8)	0.316
Low birth weight	21 (47.7)	29 (50.0)	55 (54.5)	0.722
Pre-term (28–36 weeks)	22 (50.0)	27 (46.6)	54 (53.5)	0.699
Stillbirth (FSB)	6 (13.6)	9 (15.5)	23 (22.8)	0.328
Stillbirth (MSB)	6 (13.6)	5 (8.6)	6 (5.9)	0.305
Intrauterine foetal death	2 (4.5)	10 (17.2)	20 (19.8)	0.064
Neonatal death	1 (2.3)	1 (1.7)	5 (5.0)	0.603

*SD* standard deviation *SOFA* Sequential Organ Failure Assessment score calculated with investigations taken within 24 hours of ICU admission *ICU* Intensive Care Unit *FSB* Fresh Stillbirth *MSB* Macerated Stillbirth.

* Foetal outcome was based on the birthweight, gestation of delivery and mortality at the time of delivery. Miscarriage is defined as pregnancy loss before viability of 28 weeks. Low birth weight- <2500g; Stillbirth classified as per physical features. The outcome of neonatal death was at the 2-week maternal follow-up.

The prevalence of dialysis was 37.4% (n = 76 95% CI, 31.1%–44.3%) with a mean number of 6 sessions of dialysis with the highest number of sessions of 28. The main type of dialysis was intermittent haemodialysis n = 75, 99.5% with 1 patient undergoing sustained low-efficiency dialysis (SLED), n = 1,0.4%. The two main indicators for dialysis were azotaemia (n = 68, 33.5%) and severe metabolic acidosis (n = 65,32.0%). The turn-around time from the time of decision for haemodialysis to the first session was a mode of 4 hours.

Perinatal morbidity and mortality was noted to be adversely affected by the KDIGO staging of the mother. The highest number of miscarriages (n = 16), low birth weight (n = 55), preterm delivery (n = 54), stillbirth (n = 29), foetal death (n = 20) and neonatal death (n = 5) were associated with KDIGO stage 3. However, no statistical significance was found with p values all >0.05. Recovery of renal function was complete in 44.8% (n = 91), partial in 20.2% (n = 41) with dependence of dialysis seen in 11.3% (n = 23). There was an association between renal recovery and KDIGO severity (p < 0.001), where a majority of the women in stage 1 had complete recovery. In contrast, most of those in stage 3 had partial recovery, and only stage 3 had dependence on dialysis, and more than a third of those who died were in stage 3. The average serum creatinine on admission of patients who were dependent on dialysis was 684.3 μmol/L with a mean of 2 antenatal visits and the main predisposing factor of moderate to severe anaemia (mean = 6.7g/dL).

The mortality rate was 23.6% (n = 48) with results in Table 3 showing that risks associated with mortality were mechanical ventilation (OR 6.4 95% CI 2.2–18.7 *p =* 0.001) and inotropic support (OR 21.9 95% CI 9.7–49.2 *p =*< 0.001). The mode SOFA score was 8 (n = 28,13.8%) with statistically significant higher mean SOFA score in those who died versus those who survived (12.6 ± 3.8 *p =<* 0.001 vs 8.6 ± 3.2).

**Table 3:** Factors associated with maternal mortality.

	Mortality		OR (95% CI)	p-value
	Yes *(n = 48)*	No *(n = 155)*		
**Haemodialysis, *n (%)***				
Yes	22 (45.8)	54 (34.8)	1.6 (0.8 – 3.1)	0.171
No	26 (54.2)	101 (65.2)	Reference	
**Mechanical ventilation, *n (%)***				
Yes	44 (91.7)	98 (63.2)	6.4 (2.2 – 18.7)	**0.001**
No	4 (8.3)	57 (36.8)	Reference	
**Inotropic support, *n (%)***				
Yes	35 (72.9)	17 (11.0)	21.9 (9.7 – 49.2)	**<0.001**
No	13 (27.1)	138 (89.0)	Reference	

## Discussion

The majority of the patients in this study were found to be referrals (95.1%), aged 26–35 years (47.3%), at 33–37 weeks gestation (33.0%) presenting in KDIGO stage 3 (49.8%). This is contrary to a study in Brazil that showed 64% of patients at stage 1 with a majority having a mean age of 29 years and a mean gestational age of 32.5 weeks [12]. Both were in the maternity ICU setting with a study size of 203 versus 172. The comparison could be attributed to the population served where KNH caters mainly to the referral of critical cases within the entire country while the hospital in Brazil serves mainly the people of Malaga region. Of note is that Brazil is considered a middle-income country with stronger health systems while Kenya despite its efforts in achieving UHC are still grappling. The disparity in the age group could also be contributed by the higher percentage of younger women in Kenya below 34 years of age (92%) compared to Brazil’s majority below 24 years (37.4%).

The level of education has been found to be a factor in development of PrAKI in a study done in Nigeria [19] showing it is more common in young pregnant women with below tertiary level education as is comparable to the combined 180 (88.6%) in this study.

Varying severity with other studies in Africa is a result of differing classifications of AKI in pregnancy ranging from KDIGO, RIFLE, AKIN and use of serum creatinine±urine output [9,20].

The aetiology of PrAKI seen to be hypertensive disorders in pregnancy (64.1% preeclampsia n = 45 eclampsia n = 85), obstetric haemorrhage (38.4%) and sepsis (36.5%) was comparable to a systematic review of PrAKI in Africa that showed preeclampsia, haemorrhage and sepsis as the main causes of PRAKI in the continent [9]. This is mainly a result of inadequate perinatal care, lack of specialized services, late referral of obstetric emergencies and poor management of pregnancy-related complications. However, this study emphasizes the evolving landscape of the aetiology of PrAKI previously attributed to sepsis associated with unsafe abortions [21] which in this study was found at n = 19 (9.3%).

Seventy-five percent of the study participants (n = 153) were diagnosed with anaemia. The aetiologies associated with PrAKI, including haemolysis associated with pre-eclampsia, volume depletion due to haemorrhage, and bone marrow suppression in sepsis, have been identified as contributing factors to anaemia. Consequently, preventive measures targeting these underlying causes are recommended over the routine provision of blood and blood products in healthcare facilities. However, from a public health stand-point, given the potential for disease progression and the risk of irreversible kidney function decline, blood transfusion may still be necessary to mitigate both the immediate and long-term consequences of PrAKI.

Within the critical care setting, the patients who underwent dialysis were found to be 37.4% with a mean of 5 sessions and a mode turn-around time from decision to initiation of 4 hours. A study in Moi Teaching Referral Hospital in Eldoret with a similar referral setting showed that 14.3% of the patients underwent dialysis [17]. This study was however looking at patients of PrAKI in patients with severe preeclampsia. Globally, research into the intervention of dialysis in PrAKI patients varies widely among LMIC from 1.7% to 88.2% owing mainly to differences in sample size and study settings with a majority having small sample sizes. Given the recent advent of dedicated maternal intensive care units in the continent, the use of SOFA scoring to predict the prognosis of obstetric critical care patients is limited. This study determined that higher SOFA scores were associated with a higher risk of progression to dialysis and maternal mortality. This is in keeping with studies that show higher APACHE and SOFA scores are associated with an increased risk of higher KDIGO stage, haemodialysis and use of critical care therapies [12]. The majority of the patients underwent caesarean section (59.1%) with an increased risk of preterm delivery (50.7%) and low birth weight (51.2%). There are limited studies on the effects of pregnancy related kidney injury and perinatal complications therefore it is difficult to assess whether these complications are directly implicated in the disease progression given that the predisposing antenatal factors like severe hypertensive disorders can also necessitate immediate and premature delivery and therefore low birth weight. Hopefully more studies will look into the exact sole impact of PRAKI on the outcome of the foetus/neonate.

Two weeks post diagnosis of PrAKI, the majority of the patients with KDIGO stage 1 and 2 had complete recovery (88.6%) those in stage 3 had partial recovery (27.7) and 11.3% of the total remained dependent on dialysis. Maternal mortality was increased in patients on mechanical ventilation (OR 6.4 95% CI 9.7–49.2), haemodialysis (OR 6.9 95% CI 2.9–16.3) and inotropic support (OR 21.9 95% CI 9.7–49.2) with an average ICU stay of 6 days. This is likely due to the deteriorating end-organ damage from ischaemia of circulatory collapse. This corresponds to studies in the critical care setting where maternal mortality was higher with norepinephrine therapy (OR = 21.21 95% CI 1.18–382.08, *p* = 0.00384) and haemodialysis (OR = 11.72 95% CI 1.69–81.32, *p* = 0.0128) [12]. A study done in KNH investigating PrAKI in the labour ward unit had a mortality rate of 0% [15].

This study demonstrated that the burden of PrAKI is underestimated in the Kenyan population and not as rare a sequela as previously thought. The majority of the patients presented in stage 3 as referrals from other healthcare facilities without access to ICU care and nephrology services. The impact of these findings is that deranged kidney function may cause irreversible kidney damage, adverse foetal outcome and maternal death.

The study had two limitations. The first is that it was a single-centre study which limits the ability to generalize the results. Despite this limitation, it can be inferred that the uptake and quality of care of integrated maternal health services in Kenya still has a long way to go in terms of preventing known PrAKI causing diseases at the lowest level of care. Thus preventing the progression from low risk pregnancy to referral to Kenyatta hospital and eventually ending up requiring intensive care therapies during the peri-partum period. Disregarding the burden that gestational hypertensive diseases and their effects on the kidney function of the pregnant Kenyan women has been demonstrated in this study to lead to adverse maternal and foetal morbidity and unfortunately mortality.

The second is that there is not yet a global consensus on a definitive means of diagnosing AKI in pregnancy given the physiological renal changes that occur. However, studies have shown that KDIGO presents a better prognostic value for mortality in critically ill patients to RIFLE criteria [22].

## Conclusion

The incidence of PrAKI is still underestimated in the Kenyan setting. Particularly in the critical care setting, it is associated with higher SOFA scores, risk of dialysis, and mechanical ventilation. inotropic support, low birth weight, premature delivery and maternal death.

This study has highlighted the importance of the utilization of proper antenatal care in the management of hypertensive disorders in pregnancy and obstetric haemorrhage. Prevention of acute kidney injury, before it reaches the point of referral and ICU admission, would greatly reduce maternal deaths related to PrAKI and its sequelae. There is much room for improvement in strengthening healthcare systems to diagnose and manage obstetric emergencies. The aim is that these findings will raise awareness of kidney disease in pregnancy and may lead to the allocation of resources for obstetric nephrology services that provide early diagnosis and management. We recommend that wide-scale studies are conducted in other populations which will ultimately inform a risk stratification tool for antenatal high-risk patients. In the interim, resources should be allocated for management on intensive care patients and dedicated Critical Care Units.

## Supporting information

S1 Data Raw data abstract on outcomes of pregnancy-related acute kidney injury: A retrospective study in the obstetric critical care unit at Kenyatta National Hospital 2020–2023. (XLS)

## References

[1] Piccoli GB, Zakharova E, Attini R, Ibarra Hernandez M, Covella B, Alrukhaimi M, et al. Acute kidney injury in pregnancy: the need for higher awareness. a pragmatic review focused on what could be improved in the prevention and care of pregnancy-related aki, in the year dedicated to women and kidney diseases. J Clin Med. 2018;7(10):318. doi: 10.3390/jcm7100318 30275392 PMC6210235

[2] Machado S, Figueiredo N, Borges A, São José Pais M, Freitas L, Moura P, et al. Acute kidney injury in pregnancy: a clinical challenge. J Nephrol. 2012;25(1):19–30. doi: 10.5301/jn.5000013 21928228

[3] KDIGO, Kidney Disease: Improving Global Outcomes (KDIGO), Acute Kidney Injury Work Group. KDIGO clinical practice guidelines for acute kidney injury. Kidney Int Suppl. 2012;2(1):1–141.

[4] Macedo E, Garcia-Garcia G, Mehta RL, Rocco MV. International society of nephrology 0 by 25 project: lessons learned. Ann Nutr Metab. 2019;74 Suppl 3:45–50. doi: 10.1159/000500345 31203299

[5] Conti-Ramsden FI, Nathan HL, De Greeff A, Hall DR, Seed PT, Chappell LC, et al. Pregnancy-related acute kidney injury in preeclampsia: risk factors and renal outcomes. Hypertension. 2019;74(5):1144–51. doi: 10.1161/HYPERTENSIONAHA.119.13089 31564161 PMC6791560

[6] Srinil S, Panaput T. Acute kidney injury complicating septic unsafe abortion: clinical course and treatment outcomes of 44 cases. J Obstet Gynaecol Res. 2011;37(11):1525–31. doi: 10.1111/j.1447-0756.2011.01567.x 21676078

[7] Joannidis M, Metnitz B, Bauer P, Schusterschitz N, Moreno R, Druml W, et al. Acute kidney injury in critically ill patients classified by AKIN versus RIFLE using the SAPS 3 database. Intensive Care Med. 2009;35(10):1692–702. doi: 10.1007/s00134-009-1530-4 19547955

[8] Vinturache A, Popoola J, Watt-Coote I. The changing landscape of acute kidney injury in pregnancy from an obstetrics perspective. J Clin Med. 2019;8(9):1396. doi: 10.3390/jcm8091396 31500091 PMC6780924

[9] Shalaby AS, Shemies RS. Pregnancy-related acute kidney injury in the African continent: where do we stand? A systematic review. J Nephrol. 2022;35(9):2175–89. doi: 10.1007/s40620-022-01349-2 35708883 PMC9700640

[10] Randeree IG, Czarnocki A, Moodley J, Seedat YK, Naiker IP. Acute renal failure in pregnancy in South Africa. Ren Fail. 1995;17(2):147–53. doi: 10.3109/08860229509026251 7644765

[11] Adejumo OA, Akinbodewa AA, Enikuomehin OC, Lawal OM, Abolarin OS, Alli OE. Pregnancy-related acute kidney injury: etiologies and short-term outcomes in a tertiary hospital in Southwest Nigeria. Saudi J Kidney Dis Transpl. 2019;30(6):1423–30. doi: 10.4103/1319-2442.275487 31929290

[12] Ferreira DP, Amorim FF, Matsuura AJ, de Sousa JL, Santana AR, de Souza JA. Pregnancy-related acute kidney injury: mortality and survival of patients treated at a maternal intensive care unit. Journal of Nephrology. 2020. 10.1007/s40620-020-00711-6 32072506

[13] Kayima J, Were A, Zahida Q. Impact of pregnancy related acute kidney injury on foetal survival: a single centre experience in Kenya. IOSR J Dent Med Sci. 2019;18.

[14] Pannu N, James M, Hemmelgarn B, Klarenbach S, Alberta Kidney Disease Network. Association between AKI, recovery of renal function, and long-term outcomes after hospital discharge. Clin J Am Soc Nephrol. 2013;8(2):194–202. doi: 10.2215/CJN.06480612 23124779 PMC3562863

[15] Kivai JM, Kayima J, Were A, Quereshi Z. Assessment Of Characteristics Of Patients With Pregnancy Related Acute Kidney Injury In Kenyatta National Hospital. Vol. 2, Journal of Kenya Association of Physicians. University of Nairobi; 2019. Available from: http://41.204.161.209/handle/11295/107142

[16] Ngayu NW. Pregnancy related acute kidney injury among women with preeclampsia at kenyatta national hospital: risk factors, progression and pregnancy outcomes. University of Nairobi; 2021.

[17] Mutwiri K, Itsura PTP. Renal complications among women with severe preeclampsia managed at moi teaching and referral hospital, eldoret, kenya. East Afr Med J. 2022;99. Available from: https://www.ajol.info/index.php/eamj/article/view/224552

[18] Gestational hypertension and preeclampsia: acog practice bulletin, number 222. Obstet Gynecol. 2020;135(6):e237–60. doi: 10.1097/AOG.0000000000003891 32443079

[19] Aghwana R, Onohwakpor E, Okoye O. Sun-167 pregnancy related acute kidney injury in delta state University Teaching Hospital, Nigeria. Kidney International Reports. 2019;4(7):S228. doi: 10.1016/j.ekir.2019.05.569

[20] Mjahed K, Alaoui SY, Barrou L. Acute renal failure during eclampsia: incidence risks factors and outcome in intensive care unit. Ren Fail. 2004;26(3):215–21. doi: 10.1081/jdi-120039518 15354968

[21] Prakash J, Prakash S, Ganiger VC. Changing epidemiology of acute kidney injury in pregnancy: A journey of four decades from a developing country. Saudi J Kidney Dis Transpl. 2019;30(5):1118–30. doi: 10.4103/1319-2442.270268 31696851

[22] Luo X, Jiang L, Du B, Wen Y, Wang M, Xi X, et al. A comparison of different diagnostic criteria of acute kidney injury in critically ill patients. Crit Care. 2014;18(4):R144. doi: 10.1186/cc13977 25005361 PMC4227114

